# Recommendations for Addressing Harm–Benefit Analysis and Implementation in Ethical Evaluation – Report from the AALAS–FELASA Working Group on Harm–Benefit Analysis – Part 2

**DOI:** 10.1177/0023677216642397

**Published:** 2016-05-17

**Authors:** Kathy Laber, Christian E Newcomer, Thierry Decelle, Jeffrey I Everitt, Javier Guillen, Aurora Brønstad

**Affiliations:** 1 Chief, Comparative Medicine Branch, NIEHS/NIH, Research Triangle Park, NC, USA; 2 AAALAC International, Frederick, MD, USA; 3 Chief Veterinary Officer, Sanofi, Marcy l’Etoile, France; 4 Department of Laboratory Animal Science, GlaxoSmithKline, Research Triangle, Park, NC, USA; 5 AAALAC International, Pamplona, Spain; 6 University of Bergen, Department of Clinical Medicine, Bergen, Norway

**Keywords:** harm–benefit, ethical review

## Abstract

International regulations and guidelines strongly suggest that the use of animal models in scientific research should be initiated only after the authority responsible for the review of animal studies has concluded a well-thought-out harm–benefit analysis (HBA) and deemed the project to be appropriate. The AALAS–FELASA working group on HBA has performed a literature review and based on this review, proposed a method for HBA. Examples of the working group’s approach are included in this report.

## Background

International, regional and national guidelines provided by the Office International des Epizooties (OIE),^1^ Council for International Organizations of Medical Sciences–International Council for Laboratory Animal Science (CIOMS–ICLAS),^2^ the European Directive,^3^ European Science Foundation^4^ and the *US National Research Council Guide for the Care and Use of Laboratory Animals*, 8th edition (*NRC Guide*)^5^ offer impetus for responsible entities to pursue harm–benefit analysis (HBA) during the ethical review process of animal experiments. Of note, none of these guidelines offers any parameters for what constitutes an appropriately rigorous HBA process.

The American Association for Laboratory Animal Science–Federation of European Laboratory Animal Science Associations (AALAS–FELASA) working group (WG) on harm-benefit analysis has defined HBA as a systematic process for assessing and comparing the harms and anticipated benefits of a particular animal study.^6^ The establishment of a systematic process for HBA is expected to ensure that all potential harms and benefits have been comprehensively and carefully considered during the ethical evaluation of the merits of an animal research investigation.^7^ This approach entails evaluating each component or procedure of a project for harm and considering the relative importance and relevance of the evidence (benefit) it potentially contributes to the hypothesis being tested.

A systematic HBA should help optimize the protection of animals from all undue and avoidable harms, improve consistency, completeness and transparency of the ethical evaluation, and result in a sound ethical justification for studies deemed to be scientifically valuable. The HBA helps formalize and structure the information needed to make an informed consensus decision on whether the benefits of performing an experiment outweigh the potential harms posed to the animals used in research and subsequently, whether the proposal should be accepted or rejected.

A review of the literature shows that several methods of HBA have been described, and current concepts of HBA are summarized in the *AALAS–FELASA working group report on harm–benefit analysis – Part 1*.^6^ Recommendations on how HBA can be addressed and implemented by responsible entities as part of the ethical evaluations of protocol/project applications was the other task assigned to the AALAS–FELASA WG, and is the focus of this report.

## Introduction

Persons responsible for the protocol/project applications must ensure that animal welfare is considered comprehensively according to current concepts of harm^6,8,9^ and also that harm is mitigated, for example by implementing 3R (replacement, reduction and refinement) actions.^10^ Harm to animals is a public concern and it is not limited to pain alone. With regard to benefits, researchers must explain in plain language what the expected benefits are and they must also explain why certain harms might be necessary to achieve those benefits. Furthermore the information relevant for HBA must be presented in a way such that reviewers can see what harm and benefit factors have been evaluated as well as see how they have been considered. This is important for transparency of the process and to clearly understand how the decision on approving or rejecting a particular project was evaluated by animal ethical committee (AEC) members.

## The AALAS–FELASA WG on HBA suggested framework and approach for HBA

The WG recommends a systematic approach to HBA by using a template to address all relevant aspects of harm and benefit. A template will have a normative impact; the researcher will know what harm factors are relevant for consideration which should help to promote refinement^10^ and, similarly, will know what expected benefits are anticipated that are in accordance with regulatory guidelines as well as in line with public perceptions on acceptable uses of animals in research. Also, standardization of the assessment approach is one way of increasing consistency in ethical assessment.^7^

In the following we describe a method of HBA using such a template. Based on literature reviews and discussions of the pros and cons with different models,^6^ we synthesized a new model for HBA utilizing components from previously published models. This approach entails the broad consideration of harms based upon the five freedoms^9,11^ and affords the consideration of a diverse spectrum of benefits. This tool should permit responsible entities to extract relevant information from research animal proposals in support of a deliberative and transparent HBA. Examples on how to evaluate harm and benefits are provided in the discussion. Examples of two mock research proposals are presented in Appendix 1 (Examples 1 and 2), and Tables 1 and 2 in Appendix 1 provide examples of how to use the model/tool presented by the Working Group.

### Framework for evaluation of harm/benefit

First, the animal proposal that is used by committees to evaluate harm/benefit should be framed in a manner that illustrates why a particular study that uses animals can be expected to be of value, and should contain the details needed to allow the reviewers to determine the harms.

To aid in defining the harms, the WG chose, as did Mellor,^8,9^ to consider harm factors that compromise animal subjects within the categories of the five freedoms^11^ (see Key in Table 1). This approach offers a comprehensive and broader view on animal harm and welfare, which we believe better safeguards the interests of the experimental animals^8^ and identifies important areas for the application of the 3Rs.^10^ The five freedoms^9,11^ are used to define the overarching harms of the study.

The benefits for the study are defined using an overarching set of domains that was derived from the literature review (see Key in Table 1). It is clear that benefits from applied, immediately translational research are easier to define than possible benefits from basic research, but the importance of the benefit is not correlated to the ease of its definition.

The WG acknowledged that ethical review committees are presently well positioned to assess ‘harms’ but may be less well equipped to conduct a benefit analysis.^12–14^ It may then be necessary to incorporate work from other review bodies such as scientific granting agencies and scientific peer/specialist review committees. However the summation of both ‘harm’ (Table 2a) and ‘benefit’ (Table 2b) tables needs to be linked in order to conduct a ‘harm–benefit’ analysis.

**The following steps define the process for HBA**:

1. Detail the harms and benefits at the top of both the harm (Table 2a) and benefit (Table 2b) tables.
2. Engage in a systematic review of how different animal, experimental, and environmental variables affect, or modulate, the harms associated with the proposed animal-based experiment. A suggested list (and definitions) of ‘modulating factors’ (MFs) for harms are identified and listed in the relevant table; however, this list can and should be adapted as needed based on project or institutional circumstances. Individual harm MFs are frequently inter-related and may overlap.
3. Once the list of MFs for harms is defined (Table 2a, column one), a brief description summarizing the critical point for analysis of each of the MFs in the context of the project is included (Table 2a, column two). For example, the housing conditions of the animals used in the project can be described, and details of the type and size of caging, and social/individual housing conditions can be included.
4. Depending on how the MF is applied in the context of the project, it may mitigate and/or aggravate the harm inflicted on the animals. While in some instances the effect may be only aggravating or mitigating, in others both effects may exist and should be considered. For example, if, under the ‘housing’ MF, the study requires that social animals be individually housed for a period of time, this would be interpreted as an aggravating factor, but if they are also provided with a very good enrichment program, with access to open areas and human contact, there would also be a mitigating effect, which would balance the final outcome for this particular MF. These descriptions should be included in the ‘mitigating effect’ and ‘aggravating effect’ columns.
5. The summary of the mitigating and/or aggravating components of each MF is depicted by a summary color or score (see Table 3). The color gradient scheme facilitates an easy and intuitive interpretation for the outcome of the MF analysis. We decided to use grades of red, indicating a heat map for the HBA: the deeper red, the ‘hotter’ the HBA is towards rejection of proposal. ‘Cold’ or white experiments or those with a hint of pink are easier to support. Numbers have intentionally not been used, to avoid the temptation of letting ‘calculation’ guide the decisions.^26^ Traffic light colors could also been used as suggested in a modified Bateson model.^15^ However we think that the green color used for acceptable experiments (low harm–high benefit) gives a false-positive impression that animal experiments are acceptable, while we think that animal experiments always raise ethical concerns, and there are just shades of acceptability based on the harm–benefit balance. As an example, if ‘species’ is used as an MF, crimson could be assigned to the use of non-human primates if a lower phylogenetic species could be substituted. Similarly for the ‘housing condition’ MF, social housing of dogs in pens with access to outdoor areas and a very good enrichment program could be assigned a white color, compared with the use of a crimson color for individual housing in small metabolic cages. If the details of the MF result in a dominant mitigating effect, the final color assigned in the ‘summary color’ column would be white, or ‘–’ if scoring is used, and a clear aggravating effect would have a score of ‘+++++’ and a low aggravating effect would have a score of ‘+’. Note, the ‘scoring system’ gives each category a discreet quantitative value that may give the misleading impression of a precise arithmetic assessment, whereas colors provide a wider spectrum that allows for a more intuitive and visual result. This is particularly important in view of the variety of MFs and the different weights that each may carry (protocol-dependent).
6. As with the harm table, the MFs for the benefit domains are defined and listed (Table 2b, first column). The MFs for benefits should help elucidate to the user the ‘what, why, how and when’ the benefit will be realized.^15^ The WG concludes that a summary color could be assigned for each MF but that individual mitigating and aggravating circumstances do not apply.
7. Once the harm/benefit tables (Tables 2a and b) have been completed, committees can visualize, from either the color or scoring system, the overarching intensity of the harm and the expected strength of the benefit, and make decisions on whether the proposal should be approved, rejected or modified. As an example, if the harm is intense, and the benefit minimal, the committee should reject the proposal, or work to implement approaches such as reduction, replacement and refinement, that would lower the harm level. If the benefit is high and the harm low the committee could, without reservations, ethically justify approval of the proposal.

**Table 1.**
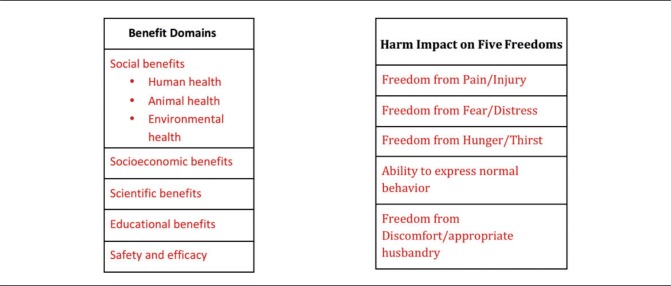
KEY: Benefit Domains and Harm Factors.

**Table 2a.**
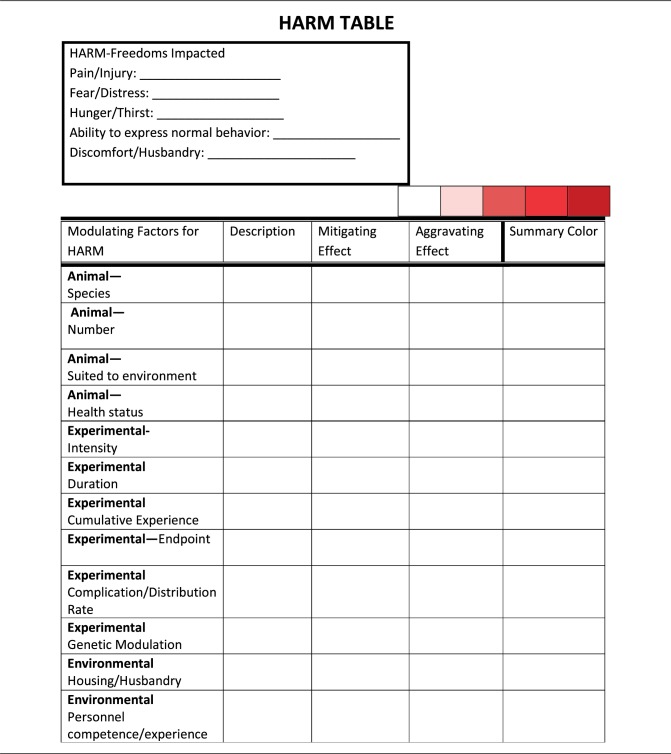
Harm table.

**Table 2b.**
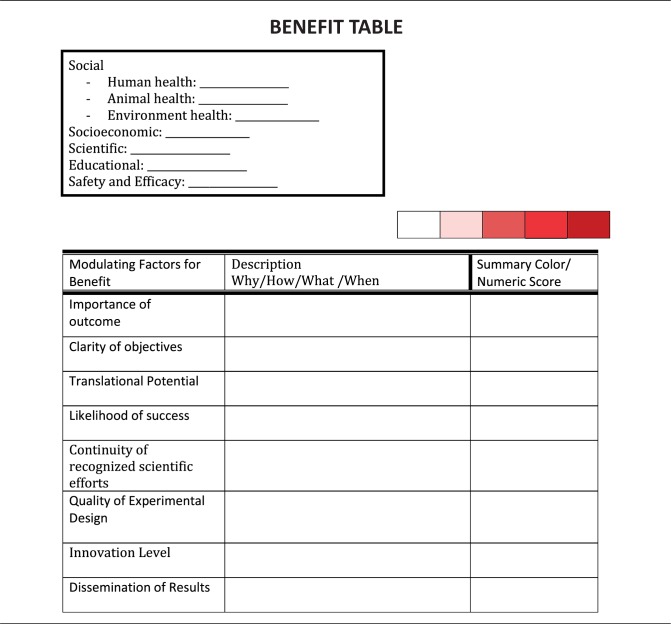
Benefit table.

**Table 3.**
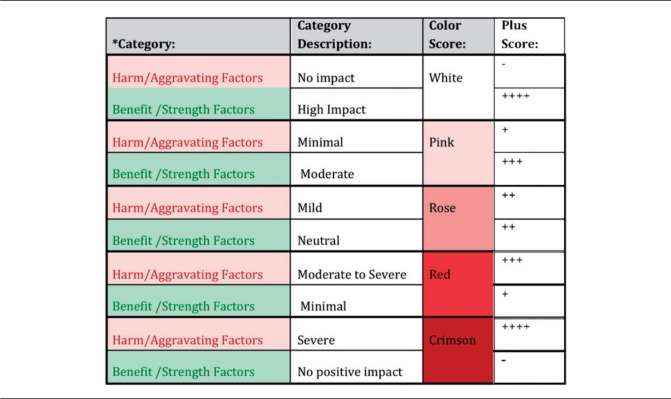
Summary of color gradient and score scheme for harm/benefit factors.

In the process of evaluating all potential harms and benefits in a systematic fashion, it is essential to realize that the weight or significance given to an individual harm/benefit will not be equal, and a single harm or benefit factor could dominate and steer the final outcome.

### Definition of MFs for harms

#### Animal

***Species***: Species proposed for the project. Potential relevant factors include: sentience, cognitive ability, phylogenetic scale, adaptation to laboratory conditions, rarity and societal concern.

***Number***: Total number of animals (by species) to be used in the project.

***Suited to environment***: Origin (source) of animals and acclimatization procedure.

***Health status***: Clinical/subclinical condition, which could cause harm to animals. Experimental and spontaneous genetic mutants that have adverse phenotypes should be considered.

#### Experimental

***Intensity of harm***: Descriptions of experimental procedures that compromise the five freedoms, and measures to alleviate them.

***Duration of harm***: Description of the immediate impact on the five freedoms and measures to alleviate them, e.g. temporary single housing of social animals.

***Cumulative experience***: Total periods of time over the animal lifespan where the five freedoms are impacted (e.g. animals that are reused for pharmacokinetic studies over their entire lifetime).

***Endpoint***: Explanations on how/if endpoints ensure animals are not subjected to unnecessary suffering, i.e. refinement, including observation procedures.

***Complication/distribution rate***: Distribution of the impact of harm among study animals and/or relative proportion of study animals subjected to different severity levels. For example, the total number of animals in a study is 100, but only 10 will be subjected to severe procedures.

***Phenotypic manipulation*:** Genetic, surgical and chemical modifications that result in impact on animal well-being as part of the experimental model.

#### Environmental

***Housing conditions***: Enclosure sizes and characteristics; social–individual housing; environmental enrichment.

***Husbandry***: Quality and provision of food, water, sanitation and identification.

***Personnel***: Competence of animal care personnel with regard to the care of the study animals, and competence of the research team with regard to the experimental procedures.

### Definition of MFs for benefits

***Purported importance of outcome***: ‘WHY’ *is the study important*? Although this cannot be defined with certainty an estimation of the importance of the outcome of the study should be made. This can be framed in terms of immediate and short-term benefits as well as the anticipated impact of the outcome for subsequent studies and long-term benefits.

***Clarity of objectives***: ‘HOW’ *will the objectives be met*? and ‘WHAT’ *will the objectives be*? The degree to which a sound hypothesis and clear objectives are elucidated can support the driving purpose of the study and ensure that the study outcome has value/benefit.

***Translational potential***: ‘WHO’ *will the study benefit*? and ‘WHEN’ *will the benefit be realized*? An assessment of how feasible the study is and how quickly the results can be expected to be applied to the benefit domain.

***Likelihood of success***: ‘HOW’ *likely is it that you will obtain the objectives desired*? In addition to the complexity and difficulty of proposed studies there are several other factors that affect the likelihood of success. These factors may include the existence of appropriate facilities, the expertise and competence of research and animal care and use personnel, as well as the level of resources and funding available to assure completion and continuity of the work. The track record of the study team should be considered in this evaluation.

***Continuity of recognized scientific efforts***: ‘WHAT’ *is the larger body of knowledge this study contributes to*? Consideration of how well this work amplifies/adds to the continuum of knowledge gained from previous studies, or indicates whether there is potential to continue to offer further benefits.

***Quality of experimental design***: ‘HOW’ *will the objectives be obtained with high quality/effective use of resources (animals, time, etc.)*? The quality of the experimental design should be considered in the benefit equation in that it ensures that the data collected are scientifically acceptable, and will validate the results obtained.

***Innovation level***: ‘HOW’ *will this study advance science beyond the specific objectives of the study itself*? Consideration of whether or not the proposed research will benefit other research through the conduct of novel and innovative processes and designs. This may include expected secondary benefits such as 3R advances.

***Dissemination of results***: ‘WHEN’ and ‘HOW’ *will the results be distributed*? How will the results be disseminated for maximum benefit? (e.g. are the results proprietary or public; presented or published, etc.).

## Discussion

### Are all harms equal?

Drawing on information from the literature^6^ and the WG professional experience, the WG offers the following points for consideration in the systematic analysis of harm.

There are inherent challenges in assessing procedural severity, and the application of professional judgment is often warranted. The level of harm is influenced by the quality of facilities, equipment, housing conditions (social versus single, quality of environmental enrichment, etc.), staff and investigator skills and competence, quality of veterinary observation and care, individual species and animal issues (phenotype and health status) as well as the definition and implementation of experimental endpoints. In summary, the level of harm is not related exclusively to the nature of the experimental procedure, but also to many other variables. Responsible entities should evaluate these elements carefully during HBA to ensure that high competence and appropriate compensatory provisions for procedures potentially impacting animal welfare and harm are applied in every instance to the fullest extent possible.

*Example: Conducting procedures in a specialized center for studies with a particular species with unique requirements may result in far less stress to the animals involved than to animals used in similar studies conducted in research facility environments without similar personnel, knowledge, expertise and equipment resources suited to the species and research investigation. Responsible authorities should ensure that essential resources and expertise are in place before allowing studies to proceed.*

The species and the behavior of the individual animal are potentially important factors when determining the level of harm. The same procedure may be scored differently depending on the species or the native reactivity and prior acclimatization. For example, a procedure conducted in a species that typically tolerates it poorly will normally be considered as more harmful than if conducted in a species that tolerates it well. Also, within a species, some individuals may be better acclimatized to experimental conditions or may have a naturally more cooperative disposition than others in behavioral studies.

*Example: Comparable stereotaxic surgical procedures in neuroscience studies can be performed in non-human primates and in rats. Although in both cases the pain associated with the procedure itself can be abolished by means of appropriate anesthesia and analgesia, the level of stress/distress created by captivity may differ by species. In addition, differences in research environment, competency of personnel (see previous examples), housing conditions, etc. may affect the final level of harm associated with the procedure.*

Consideration of harm should take into account the cumulative experience of the research animal in the research facility as well as in experimental procedures.

*Example: Responsible entities may wish to develop specific guidance concerning repetitive procedures they will allow in animals that are maintained for specific purposes. For instance in colonies of animals instrumented for safety pharmacology studies significant factors that impact harm include: how long would animals be maintained, how many drugs would they be exposed to, and how long would they be ‘rested’ between procedures. The harm to animals would not be solely defined by the experiment proposed but also by the background of the use of the research subject.*

Harm analysis should balance individual animal needs with those of the entire experimental group cohort used for data acquisition. Responsible entities should be receptive to assessing whether procedures of a greater severity to a few individuals are warranted instead of conducting procedures of less severity to a greater number of individuals in certain circumstances.

*Example: Maintenance of a small colony of cannulated animals for repeated metabolism studies subjects a small number of animals to surgical procedures and repeated doses of compounds and procedures to which they become well adapted. This might result in less cumulative harm than would occur if multiple animals were used in single experiments for which the individual bleeding procedures were more stressful and for which they were not as well adapted.*

For all animals, including genetically-modified animals, harm should be assessed using observation and scientific measures of pain and distress that occur in the research subjects and not by the assumption that alterations from the natural or wild state are deleterious a priori. Genetic modification does not necessarily result in experienced harm per se.

Harm should be assigned at the level of the whole research proposal submitted to the oversight body and should encompass all experimental procedures/conditions that potentially impact animal well-being.

There should be a mechanism of evaluating the actual level of harm regularly during the development of the protocol/project. If the harm seen is different from the prospective harm assessment, responsible entities should re-evaluate and take appropriate action. Moreover, responsible entities cannot reliably be expected to achieve a sound review process if the assessment is entirely conceptual and is limited to just a paper review.

Pilot studies are useful for determining the in vivo experimental approach and types of procedures to optimize data collection. Sometimes, however, very little prior knowledge is available to predict expected outcomes and the harm experienced by the animals. Thus, special attention in the evaluation and the actual conduct of pilot studies should be provided.

Humane endpoints can reduce the level of pain and negative impacts on the five freedoms. In some cases, researchers need some preliminary data to determine effective, early endpoints. The capacity to define early endpoints clearly impact the HBA.

### Are all benefits equal?

At a cursory glance, benefits of improving health caused by serious diseases are easy to support.^16^ However, the nature of the underlying cause of a specific disease can be an issue for discussion.^16^ Is the disease caused by predetermined factors (e.g. genetics), factors out of the individual’s control (e.g. contagious disease, intrauterine environment or exposure, or accident), or is it a consequence of a certain lifestyle (e.g. smoking) where one might expect the patient to have some influence on the outcome, or is it a result of another deterministic factor? For many types of disease there are no discrete and identifiable influences but rather undefined and complex mechanisms that are at play, and caution must be taken not to categorically devalue benefits for diseases that have a ‘lifestyle related’ ethology.^17^ Animal experiments are usually used as one tool, together with in vitro methods, epidemiological studies, clinical research or other scientific approaches. Therefore, in such cases the HBA should consider the use of animals as an additional factor in the approaches used to improve health.

Questions can be raised regarding routine product testing. Is the product of substantial importance for the improvement of the consumer’s quality of life or is the product being developed to satisfy human pursuit for luxury items or for vanity reasons? This distinction is not always clear. Product testing clearly is designed to protect health, and some products are used for several purposes, for example the botulinum toxin is used both for treating wrinkles as well as for treating neurological diseases. The European Commission has already limited animal use in favor of alternatives in some circumstances through the registration, evaluation, authorization and restriction of chemicals (REACH) legislation^18^ Also, placing restrictions on the commercial or intellectual freedoms involved in pursuing new drugs to improve performance and reduce side-effects is difficult, and few would propose defining a minimal increment of improvement necessary to justify the use of research animals in drug development. Responsible entities will undoubtedly be faced with assessing difficult ethical quandaries akin to the above on an individualized basis.

Scientific discovery efforts are often met with failure and the communication of these failures should be encouraged and facilitated and reported to ensure that negative findings bear some benefit. The publication of negative results may be regarded as counterproductive and a waste of time, potentially stigmatizing the laboratory and the research sponsor, and drawing all possible sources of failure of the laboratory into question. However, negative results are highly relevant because they reveal important knowledge and may prevent subsequent unproductive or poorly conceived inquiries in the same area, subjecting additional animals to futile experimentation. According to Claude Bernard, the founder of modern medicine, ‘there are no unsuccessful experiments … the results are always the true consequence of the conditions of the experiment’.^19^

The likelihood of success is a relevant dimension in the discussion of benefits. This does not relate to the uncertainty implicit in basic research, but to what extent the experiments are based on good scientific principles, a clear hypothesis and problem formulation, systematic review of existing knowledge, selection of appropriate methods, and research design to generate reliable data. Also, the likelihood of success depends on the research group’s expertise and available resources (knowledge, skills, personnel facilities, etc.). Likelihood of success or quality of the experiments using the chosen methods and models was presented as a separate dimension from harm and benefit in the Bateson cube model.^20,21^ We found it appropriate to discuss this under the likelihood of achieving the desired benefits and as an MF among the benefits, but did not give it its own dimension. Failure in design can lead to an unnecessary use of animals, publication of invalid results, and subsequent experiments being based upon flawed hypotheses.

There are enduring approaches to the systematic analysis of scientific problems and their investigation. The analysis logically begins with the systematic review of scientific literature relevant to the problem of interest.^22–24^ Such literature reviews should be structured, thorough and transparent. Once the problem in question and experimental hypothesis are clearly formulated against the backdrop of a thorough literature review, the strategic selection of methods may proceed. This is important because any harm to animals can only be justified if it is really necessary to answer the question. This approach to the conduct of research was emphasized by Bernard^25^ who stated, ‘Like investigations in any field of science, the merit of animal experiments ultimately depends on rigid adherence to principles of the scientific method.’

Animal research projects funded through public resources and foundations are usually subjected to the critical, peer review of the research by experts in the field who declare their independence and absence of any conflict of interest in the conduct of their duties. The WG believes that peer review of this nature may constitute a factor important to a project’s benefits and may serve as the nucleus of the responsible entities’ final evaluation of benefit in the HBA process.

### Simplied HBA

HBA using the approach described here can be a time and labor-intensive task. Therefore, responsible entities may wish to prioritize experiments according to the intensity of review and analysis deemed appropriate. A simplified Bateson square^20,21^ can be used to devise a ‘quick and simple’ way to sort experiments. Animal experiments can be simply categorized as ‘low harm–high benefit’, ‘low benefit–high harm’, ‘low harm–low benefit’ or ‘high harm–high benefit’. The low harm–high benefit experiments elicit minimal controversy. Terminal procedures (animals anesthetized for the whole experiment and then killed under anesthesia) for a beneficial purpose experience minimal harm, assuming that high standards of care are addressed. However, sacrificing a large number of animals in an experiment of this type would still raise ethical concerns if adequate scientific justification for high animal numbers were lacking. Experiments categorized as low benefit–high harm might also be easily decided. Very likely such experiments would not be ethically justified and the application would be rejected. Review of such a research proposal should prompt greater clarity on the nature and urgency of the expected benefits and should focus on reducing harm (refinement/3R). Experiments defined as low or moderate harm–low benefit or high harm–high benefit might stimulate the most discussion. Should the responsible entity accept the justification for an experiment where the benefit is poorly defined, even if harm for the animal is trivial? Experiments that are obviously beneficial but that also cause much harm are also difficult to assess. If newly emerging severe diseases occur, this might cause an urgent specific research need. Because experience with a new disease is limited it may involve some trial and failure before a research model is adequately refined to reduce harm. Finally, perhaps a majority of animal experiments (and animals) fall into a gray zone of uncertain potential benefit and experience harm levels that are not severe. In some instances, it may be permissible for responsible entities to reduce or waive the HBA review requirements in studies of this nature.

### Responsibility for HBA Outcome

The model suggested in this presentation does not say anything about who should take part in the HBA and make the final decision. However, as subjective opinions influence our evaluations^27–30^ we think a broad representation of competent persons is the only way to give a balanced HBA process and decision. This applies both for the harms and the benefits.

The WG favors and recommends the use of consensus rather than voting for the decision method of the responsible entities in conducting HBAs. The responsible entities should be able to project transparency in how they evaluated harm and benefit and how they reached their final conclusion. This transparency will aid external review by outside groups if warranted, improve information exchange, and build broader, more informed consensus and aid communication on why animals are used to the general public.

Reaching an agreement about the relevant parameters of harm and methods to palliate them is far less challenging than reaching an agreement on what constitutes a meaningful benefit resulting from a research animal study. This is clearly evident in the chart summarizing the consideration of harms and benefits in the literature for research animal studies in which the two most cited categories, benefits to humans and the quality of the research, are identified as pertinent.^3,21,26,31^ Identified benefits to animals, benefits to the environment, and knowledge benefits are also important considerations.^3,26^

The WG contends that the researchers must be able to define and describe some primary benefits for their inquiry while recognizing that there is no guarantee that projected benefits will be realized. Communications from both scientists and members of responsible entities with the WG have emphasized that the definition and assessment of benefits, particularly tangible benefits, can be a daunting and unsatisfying task in some studies. They have argued that for scientific projects that have undergone authoritative, external scientific peer review successfully and been deemed worthy of support with public funding, this should constitute an adequate, if not definitive, statement of the project’s benefits. This approach should help address the growing administrative burden that scientists face from the responsible entity overseeing research and expedite decision-making. However, unless harm to animals has also been carefully addressed in the peer review process evaluating benefits, the conclusions of the external peer review may be brought into question and the ethical implications of animal use for the specific project has not been addressed.

Finally it is important to recognize that the final comparison and evaluation of HBA will be influenced by both attitudes and competence of those making the decision.^32^

## Conclusion

The AALAS–FELASA WG on HBA has presented a model for conducting a broad, inclusive and transparent HBA. Impact on the five freedoms has been used to assess harm as this approach incorporates most of the harm parameters identified in the research animal literature and should serve other responsible entities in the thorough evaluation of harm. The central benefits encompass the advancement of human and animal health, knowledge and safety protection for humans, animals or environment. We recommend using standard qualifying questions like ‘who, what, when and how’ to help define how benefits will be realized.

Although there are ways to grade harm and benefit presented both here and by others, there is no common ‘currency’ or value system for comparing the different realms of harm and benefit. Therefore, HBA remains intractably context-dependent. The complex moral issues inherent in some HBAs are resistant to convenient automated decision making by use of algorithms and decisions will depend on individuals’ moral consciences and value judgments concerning harms and benefits for a particular project. When implementing HBA the responsible entities should be represented by different stakeholders to give a balanced evaluation and a group consensus should be the desired outcome.

## Figures and Tables

**Appendix Table 1.**
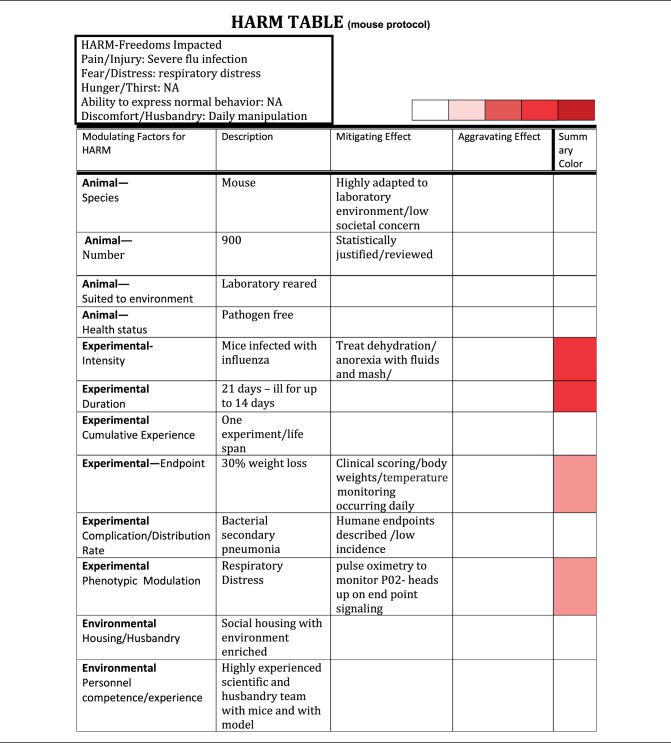
A suggestion of harm-benefit analysis for the mouse example using the WG's suggested model.

**Appendix Table 2.**
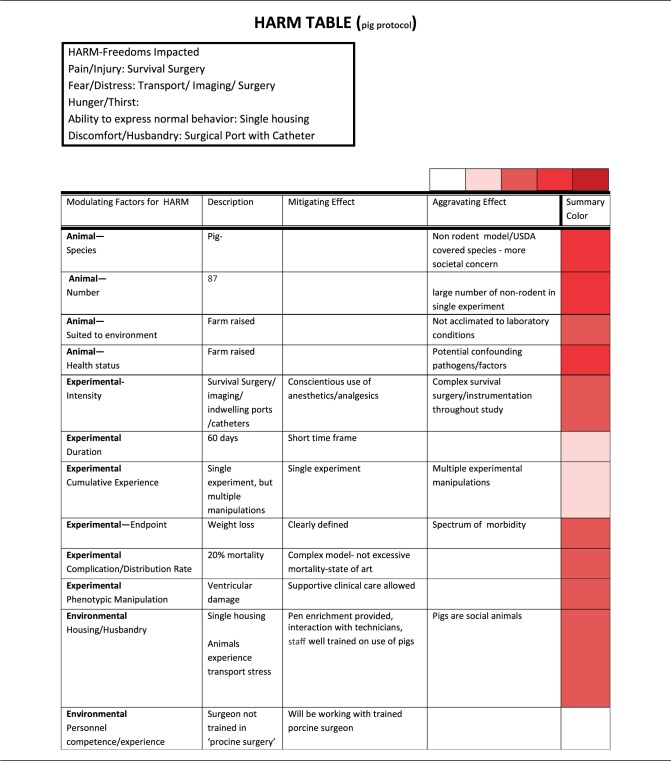
A suggestion of harm-benefit analysis for the pig example using the WG's suggested model.

## Appendix 1: Examples of applying the tool

### Protocol Example 1: Influenza infection and treatment with new chemical entities in mice

#### Description

Influenza virus (IFV) infection continues to be a significant unmet medical problem, requiring hospitalization of infants; immunocompromised, transplant and elderly individuals; as well as individuals with chronic obstructive pulmonary disease (COPD) and asthma. There are approved antiviral medications (e.g. Relenza, Tamiflu) on the market, but these only work if they are given very shortly after onset of symptoms and there is no effective therapeutic treatment for IFV. These studies will be used to evaluate new compounds with the hope of preventing and treating IFV infection. Prior to proposing this efficacy work we conducted a pilot study to determine the peak pathology associated with the viral dose given. Animals were monitored for up to 21 days to monitor weight loss and determine if they began to gain weight and recover from the infection. By day 14 the animals in the 25 TCID_50_ group had generally recovered back to normal, as predicted from studies reported in other publications. By day 10, the 125 TCID_50_ group was euthanized because they hit the 30% weight loss criteria. As we hoped, the 25 TCID_50_ group started to recover on day 10 and returned almost to normal. This provided us with two robust models to test our compounds: a severe acute infection and pathology, as well as a recovery model to test improvements in clinical scores (oxygen saturation and body weight, etc.) over time.

##### Experimental Objectives

Mice will be infected with IFV by intranasal administration and monitored daily for clinical symptoms of disease, including weight loss and lethargy. New chemical entities (NCEs) will be dosed prophylactically or at various time points post infection to identify therapeutically active compounds that will reduce viral load and/or inflammation. Blood will be drawn at various times after dosing NCEs to verify pharmacokinetic (PK) properties. Mice will be euthanized at time points identified as near the peak of virus replication and/or inflammation to determine the activity of the NCEs in preventing viral replication and/or reducing lung inflammation. Clinical scores, lung inflammation, and viral load will be used as primary endpoints to determine efficacy of the NCEs.

#### Animals

Nine hundred Balb/c mice will be used.

#### Animal justification

There are no valid alternatives to the use of animals for studying the full course of IFV disease, including both viral replication and lung immunopathology. The mouse is a highly validated animal model used in IFV research. Whole animal models are necessary for prediction of the effects of the integrated matrix on the analytical outcomes, binding of novel compounds in animals, and interaction with the virus and immune system in vivo. Mice infected with IFV develop significant lung inflammation and viral replication, which closely resembles the intended human patient populations.

#### Number justification

A typical efficacy study comprises up to 60 animals at a time. Animal numbers for the studies per group/endpoint (*n* = 5–8) have been obtained from the literature, consultation with academic labs, and previous studies using a similar influenza mouse model. Two statisticians with experience designing infectious disease studies have been consulted to determine power calculations for endpoint readouts. Separate mice will be used in each group for lung histopathology and bronchoalveolar lavage fluid (BALF) analysis since we have demonstrated in previous studies that BALF washing the lungs may alter the pulmonary histopathology and scoring. Upon completion of the pilot and initial studies, animal numbers per group will be re-evaluated with a statistician and an update will be given to the institutional animal care and use committee (IACUC) if it is deemed that lower animal numbers will be sufficient. Sixty mice per study × 15 studies per year will equal 900 mice in total. Generally, 3–6 dose groups are used for each study, which depends on the number of doses, compounds, and formulations evaluated within each study. A typical study is designed as follows:

Group 1: Mock + vehicle
Group 2: IFV + vehicle
Group 3: IFV + NCE 1
Group 4: IFV + NCE 2

Fifteen efficacy studies per year are planned.

### Assigned methods and procedures

#### Viral infection

Mice will be identified by ear punch or tail tatoo/marking. For infection, the mouse will first be lightly anesthetized in an isoflurane chamber with 1 L O_2_: 3% isoflurane. A drop of the specified volume (50–100 uL) of IFV (10–2 × 10^6^ TCID_50_) will be pipetted into the animal’s nostril, allowing the drop to be inhaled. The process may be repeated using the other nostril (usually six drops: three per nostril = one dose). Bio Medic Data Systems (BMDS) IPTT300 microchips will be implanted by subcutaneous (SQ) injection to aid in identification and body temperature monitoring, and a drop of tissue adhesive at the trochar injection site will be applied.

#### Administration of NCEs

##### Clinical effects

Drug administration, e.g. (oral or parenteral), dosages used will generally be in line with those considered pharmacologically relevant in patients. Previous PK studies will have been performed for tool compounds and other NCEs that will be used for the selection of doses for these efficacy studies, in an effort to minimize the risk of pain or distress to the animals. Regardless of the dose level administered, the animals will be closely monitored for any changes in behavior.

##### Experimental design

Groups of mice will be infected with IFV by intranasal administration and monitored daily for clinical symptoms of disease, including weight loss and lethargy. NCEs will be dosed prophylactically or at various time points post infection to identify therapeutically active compounds that will reduce viral load and/or inflammation. Blood will be drawn at various times after dosing NCEs to verify PK properties. Animals may be bled periodically during the study period to check for drug concentration levels and inflammatory markers. Typically, mice will be bled by tail nick or tip amputation and blood will be collected in a capillary tube. The blood volume collected will be under 15% in a 24 h period based on a collection of 1.0–1.8 mL total volume collected from mice in a terminal bleed. Mice will be euthanized at time points identified as near the peak of virus replication and/or inflammation to determine the activity of the NCEs in preventing viral replication and/or reducing lung inflammation. Animals targeted for terminal blood collection and/or tissue collection will be euthanized by exsanguinations under deep isoflurane anesthesia.

##### Effects of virus infection

- Change in behavior expected to be noted: depression, lethargy, reduced activity, abnormal vocalization, aggression. Influenza-infected mice may become lethargic, display reduced activity, reduce grooming causing ruffled fur, and have labored breathing. Mice will be monitored daily to observe these clinical signs of distress. Mice will be weighed once daily and their activity will be noted once they are taken out of the cage and placed onto a scale.
- Decreased feed consumption that could result in weight loss, lethargy, decreased fecal output, etc.
- Decreased water consumption that could result in dehydration, metabolic imbalance, and decreased urine output. Hydrogel will be provided on the floor of the cages if dehydration is noted.
- A validated clinical scoring system will be used to assess mice daily.
- Daily body weight will be taken. Weight loss (10% or more) or thin body condition (score 2/5 or less) will result in nutrient gel being added to the floor of cage.

##### Humane endpoints

- Cardiovascular disease with related clinical signs (e.g. coughing, respiratory distress, cyanosis, limb edema).
- Hunched posture in conjunction with other clinical signs and especially if debilitating or prolonged (3 days).
- Inability/unwillingness to ambulate to reach food or water.
- Marked changes in behavior noted: severe depression, non-responsiveness, listless, unwilling to move.
- Other clinical signs judged by experienced technical staff to be indicative of morbidity or being in a moribund condition.
- Weight loss of up to 30% is anticipated. We are requesting IAUC permission to keep mice alive for up to 30% weight loss, instead of 20%, so we can monitor the full course of infection and disease and allow for therapeutic treatment of lung inflammation. Publications using the strains of IFV in this protocol have documented weight loss of up to 35% with higher doses of virus, but we plan to use lower doses and keep weight loss at 30% or below. Mice also have been documented to recover body weight over time if they are monitored for up to 21 days. We aim to determine an appropriate course of disease to induce significant lung inflammation and virus replication to allow for therapeutic treatment with NCEs, but without causing severe disease or weight loss of >30%.

##### Housing and husbandry

Mice will be housed in microisolator caging, three per cage, in the pharmaceutical company vivarium. Cages will be provided with nesting material and plastic huts for warmth and environmental enrichment. The ambient temperature of the room will be raised and controlled at 75 ± 2°F. This is an Association for Assessment and Accreditation of Laboratory Animal Care (AAALAC) International accredited animal care and use program. The rodent housing facility is excellent and supported by an experienced (>5 years average) animal care staff, all of whom are certified by American Association for Laboratory Animal Science (AALAS).

### STUDY OBJECTIVES

The development and progression of congestive heart failure has reached epidemic proportions worldwide. It is estimated that currently over 10 million patients suffer from this condition. One of the common causes for congestive heart failure are the long-term effects of a heart attack – myocardial infarction (MI). Despite significant advances in our abilities to reopen and restore blood flow to the heart muscle, methods to prevent the long-term effects of the damaged myocardium have not been forthcoming. A structural milestone in the development and progression of congestive heart failure secondary to MI is myocardial remodeling. This is defined as changes in left ventricular (LV) geometry and structure which in turn can reduce pumping efficiency. It is now recognized that the region of the myocardium surrounding the MI changes shape and size and that this in turn is translated into overall changes in LV geometry. This phenomenon is termed ‘infarct expansion’ and has been identified as an important therapeutic target to minimize post-MI remodeling, subsequent LV remodeling, and in turn reduce the progression to heart failure. Exacerbated infarct expansion in the early post-MI period has been hypothesized to be an independent predictor for accelerated LV dilation and, potentially, progression to the development of heart failure post-MI. Accordingly, the goal of this study will be to longitudinally measure regional and global LV geometry in the same post-MI pigs to develop a relationship between early regional changes in infarct geometry (regional infarct expansion) and later increases in LV dimensions (global LV remodeling).

This study will use MRI in a well-established porcine MI model to develop a temporal relationship between regional and global changes in LV geometry in the same pigs post-MI, we believe we will be contributing new information using MRI.

### RATIONALE FOR ANIMAL USE

1. *Explain your rationale for animal use*. There are no in vivo models that simulate regional and global remodeling in the LV following MI. Therefore, there are no alternatives to performing these procedures in animals.
2. *Justify the appropriateness of the species selected*. Pigs have been shown to be an excellent model for performing studies to determine changes in the myocardial extracellular matrix (ECM) in a number of simulated cardiac disease states. Importantly, it has been demonstrated that pigs most accurately reflect the coronary anatomy of humans and respond in a similar fashion to myocardial ischemia/infarction. Secondly, pigs can be obtained in consistent sizes and weights and therefore, reducing variability between experimental observations.
3. *Justify the number of animals to be used*. Power analysis indicates 10 animals in the non-MI group for Phase 1 and 10 animals in the MI group for Phase 2. We anticipate a mortality rate of 20% so will assign 13 animals per group with the studies repeated in triplicate. A total of 87 animals will be used, 29/year/3 years.

### EXPERIMENTAL DESIGN AND ANIMAL PROCEDURES

Pigs will be allowed to acclimatize within university facilities for a minimum of seven days. For the first five days that the pigs are in-house, all pigs will be administered erythromycin (250 mg PO, TID) due to their conventional health background. On the day prior to instrumentation, on the day of instrumentation, and the day following instrumentation, the pigs will be administered Naxcel (3.0 to 5.0 mg/kg, intramuscularly).

#### Surgical modeling

For control animals, a purse string will be made in the thoracic aorta and a catheter connected to an access port will be advanced to the aorta at the level of the diaphragm. The access port connected to the catheter will be placed in a subcutaneous pocket and secured in place using silk ties. For MI-induced animals, a pericardiectomy exposing the LV free wall, the left atrium, the circumflex artery and the obtuse marginal (OM) branches will be performed. A purse string will be made in the thoracic aorta and a catheter connected to an access port advanced to the aorta at the level of the diaphragm. The access port connected to the catheter will be placed in a subcutaneous pocket and secured in place using silk ties. OM arteries from the circumflex coronary artery will be identified. Ligatures (Proline 4.0) will be placed around the origins of OM1 and OM2. MI will be induced by permanent ligations of OM1 and OM2. Both control/MI pigs will be imaged at 28, 42, and 56 days post-surgery and terminally studied after imaging at 56 days post-surgery.

#### Serial imaging measurements

##### Sedation and anesthetic induction

Animals will be sedated with benzodiazepam mixed in a food administered 2 h prior to study, and placed in a custom-designed sling that will allow the animal to rest comfortably. The animals will be intubated with a cuffed intubation tube and will be allowed to self-ventilate with 0.5–3.0% isoflurane delivered through a portable anesthesia machine. Heart rate and rhythm will be continually monitored using surface electrocardiogram (ECG) recordings.

The skin over the vascular access port on the back will be shaved and prepared in a sterile fashion with alternating wipes of betadine and alcohol. The access port will be entered with a custom needle (Huber) and drawback of arterial blood confirmed. The access port with the associated intravenous line will then be capped using sterile supplies and housed in a custom-designed pouch.

##### Imaging

Longitudinal measurements of LV geometry and function will be performed using MRI. As an additional measure of perfusion, gadolinium (Gd) enhanced contrast MRI images will be recorded.

##### Recovery

Following the completion of imaging studies, the animals will be transported back to their housing facilities. The access port will then be flushed with heparinized saline (1000 U/mL) supplemented with cefazolin. The Huber needle will be removed. The animals on which future imaging studies will be performed will be weaned off anesthesia, extubated, and then returned to cages once recovered from anesthesia. The animals in which the final set of imaging studies are completed, will be processed for terminal studies.

##### Terminal studies

Following the final set of MRI studies at 56 days post-MI, the pigs will be anesthetized for assessment of global and regional LV functions, microdialysis measurements and hemodynamics. Anesthetic induction will commence using isoflurane (3%) in a mixture of oxygen and nitrous oxide (67:33%, 1.5 L/min) delivered by face mask. Once the pig is adequately anesthetized, peripheral venous access (ear) will be obtained and a 2 µg/kg dose of sufentanyl (ESI) will be injected through the ear vein cannula. A 0.1 mg/kg dose of etomidate and a 10 mg dose of vecuronium will then be administered intravenously after ensuring that the animal remains adequately anesthetized. This results in a rapid deepening of the already established surgical plane of anesthesia. An endotracheal tube will be surgically placed via a midline submandibular incision and mechanical ventilation established. Anesthesia will be maintained throughout the procedure by delivery of 0.5% isoflurane and intravenously administered morphine (ESI; 3 mg/kg/h). The delivery of isoflurane, nitrous oxide and morphine will be titrated to maintain stable physiological hemodynamic and respiratory profiles. Intensive and continuous monitoring of various vital signs will provide the necessary means to ensure a complete and stable surgical plane of anesthesia. Following stabilization of this surgical anesthetic plane, an intravenous infusion of vecuronium (15 mg/h) will be initiated. This infusion will be titrated as needed to provide continuous muscle relaxation which facilitates appropriate mechanical ventilatory control and a stable surgical field. Additional 5 mg boluses of vecuronium will be administered as needed to support these goals. The heart rate and blood pressure will be carefully monitored to ensure that the animals remain at a stable surgical plane of anesthesia throughout the procedure.

A multi-lumen thermodilution catheter will be positioned in the pulmonary artery via the right external jugular vein. An 8 F introducer with a side-arm will be placed in the right carotid for blood pressure measurements and arterial access. The aortic access port will also be connected to monitor systemic pressures. A Foley bladder catheter will be surgically placed and secured via a midline suprapubic retroperitoneal incision. A sternotomy will be performed and a vascular ligature will be placed around the inferior vena cava in order to perform transient caval occlusion. A previously calibrated microtipped transducer will be placed in the LV through a small apical stab wound. Piezoelectric crystals will be positioned in the LV endocardium in order to provide an orthogonal myocardial dimension across the short axis in two regions: the MI region and the remote region. The remote regions will be defined as the area served by the left anterior descending artery (LAD).

The terminal procedure is expected to take place over an 8 h period.

### OTHER

- **Transportation** For imaging studies, animals will be transported to the magnetic resonance imaging (MRI) facility in an enclosed van. Animals will be loaded and unloaded from the vehicle in such a manner that visibility to the public will be minimized through careful selection of transport route and utilization of enclosed loading bays. This vehicle will be specifically outfitted to transport the sling and anesthesia machine (with associated monitoring equipment) in a locked position so that the animals being transported remain stationary relative to the vehicle. In addition, a high capacity power inverter will be installed to provide power to the anesthesia machine and monitoring equipment while in the transport van. For terminal studies, animals will be transported to the investigators laboratory in a large mobile animal crate covered with a sheet.
- **Animal identification methods**

Pigs will have ear tags placed upon arrival.

- **Methods of restraint**

Pigs will be restrained in a pig sling for a period of up to 60 min. This is not considered prolonged, and the pigs appear comfortable in the sling. In our experience, no acclimatization is necessary.

- **Experimental injections or inoculations**

Gd-DTPA will be manually injected at a dose of 0.1 mmol/kg through the vascular access port for imaging.

- **Blood withdrawals**

In order to provide an estimate of MI size, an aortic blood sample will be drawn from the subcutaneous access port. The pig will be placed in a custom-designed sling that will allow the animal to rest comfortably in a non-restrained fashion. The area around the access port (5 cm) will be washed and prepped and a sterile field created. The access port will be entered with a custom needle (Huber) and 3 cc of aortic blood drawn. The access port will then be flushed with heparinized saline (1000 U/mL) supplemented with cefazolin. This entire procedure will last approximately 30 min. Throughout the 56-day study period, additional 3–5 cc blood samples will be collected weekly to assess clinical chemistry and complete blood count (CBC) parameters.

- **Food or fluid restriction**

Animals will be fasted for 12 h prior to the surgery.

- **Pharmaceutical-grade and non-pharmaceutical-grade compounds**

Not available.

- **Resultant effects**, if any, that the animals are expected to experience.

Animals are expected to experience hemodynamic compromise that may result in inappetance, inmobility, weight loss, vomiting, diarrhea. Pain and distress are expected with the surgical procedures.

- **Other potential stressors** [*e.g. noxious stimuli, environmental stress*] **and procedures to monitor and minimize distress**

The access port can create a level of discomfort/distress for the pig. We will evaluate its patency weekly, and evaluate daily for signs of infection.

- **Experimental endpoint criteria** The animals will be pulled from the study and euthanized if their weight loss exceeds 20% of their starting weight.
- **Veterinary care**

Every surgically-altered animal involves a large resource commitment, so every effort will be made to address clinical issues that arise with the vascular access port, hemodynamic compromise or other issues. Investigators will consult with staff veterinarians for a treatment plan.

### SURGERY

If surgery is proposed, complete the following:

1. *Identify and describe the surgical procedure(s) to be performed. Include preoperative procedures* [*e.g. fasting, analgesic loading*], *and monitoring and supportive care during surgery. Include the aseptic methods to be used.* The animals will be fasted for 24 h, and on the morning of surgery, a 100 µg fentanyl patch (5 µg/kg/day, release rate of 50 µg/h) will be applied in addition to an intramuscular injection of buprenorphine (0.05–0.1 mg/kg). A surgical plane of anesthesia will be provided through the use of inhalation isoflurane. Just prior to surgery, anesthesia will be induced with ketamine (22 mg/kg), acepromazine (0.04 mg/kg), and atropine (0.04 mg/kg) by trained staff and the pigs will be placed in a custom-designed pig sling. An ear vein will be accessed and the venous cannula left in place to administer intravenous fluids (e.g. lactated Ringer’s) and other pharmacological agents (e.g. antiarrhythmics, such as lidocaine) if needed. An ECG and pulse oximetry will be established. Anesthetic induction will also be established using a face mask delivering isoflurane (3%, 1.5 L/min) and nitrous oxide (0.5 L/min). The animal will then be intubated with a cuffed endotracheal tube and ventilated at a flow rate of 22 mL/kg/min. Regulation of the delivery of isoflurane will be used to maintain a stable heart rate and blood oxygenation, and will be increased if either of these parameters rises by over 10% from ambient levels. Oxygen saturation and heart rate will be monitored continuously to provide a sensitive means to ensure a complete and stable surgical plane of anesthesia. A lidocaine infusion will be initiated with a 3 mg/kg bolus followed by a constant infusion of 120 mg/h.
2. *Identify the individual(s) who will perform surgery and their qualifications, training, and/or experience.* Dr Expert’s fellow will be performing the surgeries, initially under the direction of Dr Expert, but practice, independently. Dr Expert is a trained cardiothoracic surgeon who will be getting training in this particular procedure in pigs from Dr Supreme, a veterinarian surgeon experienced in CV procedures.
3. *Identify the location where surgery will be performed*. Dr Expert has her own surgical facilities in Building D and surgeries will be conducted in that location.
4. *If survival surgery, describe postoperative care that will be provided and frequency of observation. Identify the responsible individual(s) and location(s) where care will be provided* [*building(s) and room(s)*]. Include detection and management *of postoperative complications during work hours, after hours, weekends and holidays.* The animal will be recovered from the surgery in an intensive care unit under the direction of the staff veterinarians. Buprenorphine 0.005–0.02 mg/kg will be utilized for immediate post-surgical pain. For prolonged post-thoracotomy pain and more prolonged analgesia, ketoralac tromethamine will be administered.
5. *If non-survival surgery, describe how euthanasia will be provided and how death will be determined*. Following completion of the protocol described above, isoflurane delivery will be increased to 5%, and maintaining full anesthesia, cardioplegic arrest will be induced through delivery of a 24 mEq potassium solution in lactated Ringer’s through the aortic root. The heart will be harvested and the LV isolated and placed in chilled Krebs solution.
6. *Are paralytic agents used during surgery? If yes, please describe how ventilation will be maintained and how pain will be assessed*. Pancuronium (15 mg/h) will be administered for the terminal procedures. The pig will be on a mechanical ventilator and blood pressure, heart rate and oxygen levels will be continuously monitored.
7. *Has major or minor survival surgery been performed on any animal prior to being placed in this study?* *If yes, please explain*. No
8. *Will more than one survival surgery be performed on an animal while in this study? If yes, please justify*. No

### PAIN OR DISTRESS CLASSIFICATION AND CONSIDERATION OF ALTERNATIVES

1. Pain or distress classification for USDA covered species. USDA Classification D = Animals subjected to potentially painful or stressful procedures for which they receive appropriate anesthetics, analgesic and/or tranquilizer drugs.
2. Consideration of alternatives:

A Pub Med search from 2012–2015 using the keywords ventricular remodeling, pig, animal alternatives indicated that alternatives were not available.

### ANESTHESIA, ANALGESIA, TRANQUILIZATION, OTHER AGENTS

#### Analgesics

*Perioperative analgesia*: 100 ug fentanyl patch (5 ug/kg/day); intramuscular injection of buprenorphine (0.05–0.1 mg/kg/ im).

*Post-operative analgesia*: beuprenorphine 0.005–0.02 mg/kg. Long-term discomfort: Ketoralac at veterinary recommended dose.

#### Anesthesia

Ketamine (22 mg/kg), acepromazine (0.04 mg/kg), atropine (0.04 mg/kg), isoflurane, nitrous oxide.

### METHOD OF EUTHANASIA OR DISPOSITION OF ANIMALS AT END OF STUDY

Pigs will be euthanized by exsanguination under a surgical plane of isoflurane anesthesia. Animals deemed to be in distress will be euthanized by a barbiturate overdose. These methods are consistent with the recommendations of the Panel of Euthanasia of the AVMA.

### EXEMPTIONS FROM ENVIRONMENTAL ENRICHMENT AND SOCIAL HOUSING

Previous work has shown that the externalized instrumentation requires individual housing, the pigs cannot be housed socially. Enrichment devices will be provided as determined by the veterinary staff.

### PRINCIPAL INVESTIGATOR CERTIFICATIONS TRAINING

1. I certify that I have attended the institutionally required investigator training course.

 Year of Course Attendance: *20xx*

 Location: *Certified Laboratory Animal Training Center*

Additional Training: Board certified Cardiothoracic surgeon

Additonal Training: Board Certified Veterinary Surgeon
